# Pre-Existing Liver Disease and Toxicity of Antifungals

**DOI:** 10.3390/jof4040133

**Published:** 2018-12-10

**Authors:** Nikolaos Spernovasilis, Diamantis P. Kofteridis

**Affiliations:** 1 Department of Internal Medicine and Infectious Diseases, University Hospital of Heraklion, P.O. Box 1352, 71110 Heraklion, Crete, Greece; nikspe@hotmail.com; 2 School of Medicine, University of Crete, 71110 Heraklion, Crete, Greece

**Keywords:** liver disease, hepatic impairment, invasive fungal infection, antifungal agent, antifungal drug, toxicity

## Abstract

Pre-existing liver disease in patients with invasive fungal infections further complicates their management. Altered pharmacokinetics and tolerance issues of antifungal drugs are important concerns. Adjustment of the dosage of antifungal agents in these cases can be challenging given that current evidence to guide decision-making is limited. This comprehensive review aims to evaluate the existing evidence related to antifungal treatment in individuals with liver dysfunction. This article also provides suggestions for dosage adjustment of antifungal drugs in patients with varying degrees of hepatic impairment, after accounting for established or emerging pharmacokinetic–pharmacodynamic relationships with regard to antifungal drug efficacy in vivo.

## 1. Introduction

Invasive fungal infection (IFI) is a leading cause of morbidity and mortality among immunocompromised and critically ill patients [1,2]. Although antifungal drug options have increased in recent years, effective management of IFI depends mainly on early and appropriate individualized treatment that optimizes efficacy and safety based on local epidemiology, drug spectrum of activity, pharmacokinetic (PK) and pharmacodynamic (PD) properties of the antifungal agent, and patient related factors [3].

Pre-existing liver disease in patients with IFIs raises significant concern about the safety of antifungal agent administration. The liver is the primary site of drug metabolism, and hepatic disease can significantly alter the PKs of antifungal drugs, mainly through impaired clearance [4]. Moreover, other variables that affect PKs such as liver blood flow, biliary excretion and plasma protein binding may be altered in patients with pre-existing hepatic dysfunction [4]. These patients may also tolerate drug-induced liver injury (DILI) more poorly than healthy individuals [5]. Furthermore, in the cirrhotic patients, drug-related extrahepatic effects, such as renal failure, gastrointestinal bleeding and hepatic encephalopathy, are more likely to occur [6]. Hepatic functional status is also an important determinant of the drug–drug interaction (DDI) magnitude due to enzyme inhibition or induction in the liver [7].

It is important to distinguish isolated biochemical injury from hepatic dysfunction [8]. In general, DILI is characterized by elevations in hepatic enzymes, resulting from the effect of an active drug or its metabolites to the liver [9]. This biochemical abnormality is not necessarily accompanied by clinically significant liver dysfunction, since liver has a notable healing capacity [8]. However, DILI can be the cause of hepatic dysfunction, manifested by hyperbilirubinemia and coagulopathy [10], or even acute liver failure, presented with jaundice and hepatic encephalopathy [11].

Liver injury induced by a drug is generally classified as either intrinsic, which is predictable, dose-dependent and reproducible in preclinical models, or idiosyncratic, which is unpredictable and dose-independent [12,13,14]. An international expert group of clinicians and scientists comprehensibly proposed the clinical chemistry criteria for the diagnosis of DILI, taking also into account the possibility of pre-existing liver enzymes abnormalities (Table 1) [15]. Furthermore, the ratio of serum alanine aminotransferase (ALT) to alkaline phosphatase (ALP), expressed as multiples of upper limit of normal (ULN), is called R ratio or value, and is used to classify DILI in individuals with previous normal liver tests into three categories: hepatocellular (*R* > 5), cholestatic (*R* < 2) and mixed (*R* of 2–5) [16]. Bilirubin, although not incorporated into the R ratio, remains an essential marker in calculating the Model for End-Stage Liver Disease (MELD) score and the Child–Pugh score [17,18]. Both these prognostic models are also used to assess hepatic function, with the Child–Pugh score being the most commonly used method in cirrhotic patients among studies submitted to the US Food and Drug Administration (FDA) although it is not associated directly with PK changes [19] and does not represent a reliable estimator of liver function [20].

The risk of developing liver injury and possible hepatic dysfunction by an antifungal agent depends on several factors. The chemical properties of the agent, demographics, genetic predisposition, comorbidities including underlying hepatic disease, concomitant hepatotoxic drugs and DDIs, severity of the illness, and liver involvement by the fungal infection, all affect the possibility for hepatotoxicity [21]. Under these circumstances, it can be difficult to attribute DILI due to antifungals to only one factor.

In general, published literature regarding the use of antifungal agents in patients with pre-existing liver disease is somewhat inconclusive. A clear understanding of antifungal-caused liver injury in patients with underlying hepatic impairment is lacking, and recommendations for dosage adjustments in these cases are not straightforward [3,22]. Most of the information about antifungal dosing regimens is derived from clinical trials and PK studies, in which only few patients with a varying level of liver impairment were included [20]. For some antifungals, a dose reduction is recommended in the manufacturers’ product characteristics in cases of pre-existing hepatic dysfunction, while for other antifungal agents no dosage adjustment is required or recommended [22].

The aim of the present review is to provide an overview of the safety profile of the various antifungal agents in patients with underlying liver disease. The intention is to summarize current data on the PKs of antifungals in these individuals and to increase clinical awareness of how various antifungal compounds should be used under these circumstances.

## 2. Antifungal Agents

The current antifungal armory for IFIs includes polyenes (amphotericin B-based preparations), flucytosine, triazoles (fluconazole, itraconazole, voriconazole, posaconazole, and isavuconazole), and echinocandins (caspofungin, micafungin, and anidulafungin) [23]. These compounds differ from each other in their spectrum of activity, pharmacokinetics/pharmacodynamics (PK/PD) properties, indications, dosing, safety profile, cost, and ease of use [3,24,25].

### 2.1. Polyenes

Amphotericin was introduced in therapy in 1958 as amphotericin B deoxycholate (AmBD), but its clinical usefulness is limited because of nephrotoxicity and infusion-related reactions [24,26]. Three lipid formulations of amphotericin B (AmB), liposomal amphotericin B (LAmB), amphotericin B lipid complex (ABLC), and amphotericin colloidal dispersion (ABCD; discontinued in most countries) were developed in the 1990s to reduce the toxicity observed with AmBD [24]. AmB interacts with ergosterol in the fungal membranes leading to the formation of membrane-spanning pores, ion leakage, and ultimately fungal cell death [27]. Additional cytotoxic mechanisms of AmB are inhibition of the fungal proton-ATPase and lipid peroxidation [28]. It is eliminated unchanged mainly via urine and feces [29]. Because of its broad antimycotic spectrum, AmB is a cornerstone in the treatment of serious and life-threatening fungal infections. The daily dose for AmBD ranges from 0.3 to 1.5 mg/kg, while the recommended standard doses for the lipid formulations of AmB are much higher [29,30]. Specifically, for LAmB the usual daily dose ranges from 3 to 5 mg/kg, but doses up to 10 mg/kg/d can be administered in cases of rhino-orbital-cerebral mucormycosis [29]. For ABLC the usual dose is 5mg/kg/d, while for ABCD the daily dose ranges from 3 to 4 mg/kg [30].

Generally, lipid-based formulations of AmB present at least the same efficacy as AmBD and are even superior in the treatment of certain fungal infections, such as mild to moderate disseminated histoplasmosis in patients with acquired immunodeficiency syndrome (AIDS), while they are associated with a safer profile [30,31,32]. Notably, in some studies, the administration of LAmB was associated with lower toxicity rates, namely infusional and kidney toxicity, compared to other lipid formulations [33,34,35]. However, differences in drug-induced nephrotoxicity between lipid-based formulations of AmB continue to be a subject of debate [36,37]. Other commonly encountered adverse effects of AmB preparations, apart from nephrotoxicity and infusion reactions, include hypokalemia, hypomagnesemia, and anemia [27,38]. Liver injury due to AmB therapy is relatively subtle and reversible, with its incidence reaching 32% for LAmB and 41% for ABLC in some clinical studies [21,39,40]. Interestingly, lipid formulations of AmB, mainly LAmB, seem to have a stronger association with DILI than AmBD, probably due to the carriers of these formulations [24,33,40,41]. In any case, clinically evident liver injury and treatment discontinuation due to AmB preparations are rare [21,27].

No specific recommendations are available for AmB preparations in the case of pre-existing hepatic impairment, but considering their limited hepatic metabolism, dosage adjustment is unlikely to be necessary [22]. Data on the PKs of AmB in pre-existing liver disease are sparse and clinical studies are lacking so far. In a retrospective single-center non-randomized autopsy-controlled study, Chamilos et al. compared hepatic enzymes elevations and histopathological findings in the livers of 64 patients with hematologic malignancies who had received LAmB or ABLC for at least 7 days, as a treatment for IFIs [42]. Among these patients, there were 22 patients with elevated liver enzymes at baseline, more than five times the ULN. None of the patients with acute liver injury, including those with abnormal baseline hepatic biochemical parameters, showed the histopathological changes induced by liposomal formulations of AmB that have been reported in animal studies [42]. Another study assessed the PK properties of ABCD in 11 patients with cholestatic liver disease compared to 9 subjects with normal liver enzymes [43]. Pre-existing cholestatic liver disease had no significant influence on steady state PKs of liberated AmB, and the authors concluded that the standard dosage of ABCD is probably appropriate for these patients [43].

### 2.2. Flucytosine

Flucytosine became available in 1968 [44]. It is taken up by fungal cells by cytosine permease and converted intracellularly into fluorouracil, which is further metabolized into 5-fluorouridine triphosphate and 5-fluorodeoxyuridine monophosphate, resulting in inhibition of fungal protein and DNA synthesis [45]. It is mainly eliminated by the kidneys, while it is minimally metabolized in the liver [46]. The high occurrence of resistance precludes its use as a single agent. Nowadays, flucytosine is used in combination therapy with AmB as first-line therapy in cryptococcal meningoencephalitis [47]. Furthermore, it may be added to other regimens for the treatment of severe pulmonary cryptococcosis, central nervous system candidiasis, *Candida* endocarditis, and *Candida* urinary tract infections [47,48,49]. Flucytosine’s recommended dosage in individuals with normal renal function ranges from 50 to 150 mg/kg/d divided in four doses for both oral and intravenous formulation, while dosages up to 200 mg/kg/d can be administered [29,50].

Flucytosine’s most significant adverse effects is myelotoxicity, mainly neutropenia and thrombocytopenia, and hepatotoxicity, and both are thought to be due to the effects of fluorouracil [46,51]. Because human intestinal flora is capable of converting flucytosine into fluorouracil in vitro, oral administration of the drug might be associated with more side effects than intravenous administration [51]. Liver injury is frequently encountered during treatment with flucytosine and the incidence varies from 0% to 41%, probably due to the different definition of liver injury in different studies [24]. The elevation in liver enzymes is usually mild to moderate and reversible on discontinuation, while two cases of severe liver necrosis have been reported in patients who received flucytosine for candidal endocarditis [46,52]. Both myelotoxicity and liver toxicity have been associated with high flucytosine concentrations in the blood. Therapeutic drug monitoring (TDM) is advisable 3–5 days after initiating therapy and after any changes in the glomerular filtration rate (GFR) to keep the 2 h flucytosine post-dose levels between 30 to 80 mg/L [53]. DDIs involving the cytochrome P450 (CYP450) pose a minor concern for flucytosine administration [29].

For patients with pre-existing hepatic impairment, limited data are available regarding the PK properties and the safety of flucytosine. In 1973, Block studied for the first time the effect of hepatic insufficiency on flucytosine concentrations in the serum of rabbits with chemically induced acute hepatitis [54]. No influence of the hepatic function on serum concentration of the drug was observed. In the same paper, a single patient with biopsy-proven cirrhosis was described as treated with flucytosine for cryptococcal meningitis. Drug concentrations in serum were measured at 1, 2, and 6 h after a dose and did not differ from concentrations determined simultaneously in 10 patients with cryptococcal infection and normal liver function being treated with the same dose of flucytosine [54]. However, given the fact that liver injury due to flucytosine treatment is a common adverse effect in many studies, this antifungal agent should be used with extreme caution or even be avoided in this patient population, although there are no dosage adjustments provided in the manufacturer’s labeling [29,50]. Combined treatment with AmB may lead to the accumulation of flucytosine because of AmB-induced nephrotoxicity, further complicating the matter [55]. In addition, a recent study examining the hepatotoxicity induced by combined therapy of flucytosine and AmB in animal models showed a synergistic inflammatory activation in a dose-dependent manner, through the NF-κB pathway, which promoted an inflammatory cascade in the liver. The authors suggested that the combination of flucytosine and AmB for the treatment of IFIs in patients with hepatic dysfunction requires careful clinical, biochemical, and drug monitoring [56].

### 2.3. Azoles

The azole antifungals are synthetic compounds that can be divided into two subclasses, the imidazoles and the triazoles, according to the number of nitrogen atoms in the five-membered azole ring [29]. The imidazoles include ketoconazole, miconazole, and clotrimazole [21]. Miconazole was at one time administered intravenously for the treatment of certain IFIs, but soon this formulation was withdrawn due to toxicity associated with drug solvent [57]. Ketoconazole was frequently applied for systemic mycoses in the past, but it is now avoided due to its liver and hormonal toxicity [23]. The triazoles consist of fluconazole, itraconazole, voriconazole, posaconazole, and isavuconazole [29]. Azole antifungals inhibit the synthesis of ergosterol in the fungal cell membrane [29]. Despite this mechanism of action, azoles are generally fungistatic against yeasts, while the newer members of this subclass possess fungicidal activity against certain molds [23,48]. At present, these agents are considered the backbone of IFI therapy [23,49,58].

The most common adverse events (AEs) with all the triazoles, and especially with oral itraconazole, are nausea, vomiting, diarrhea, and abdominal pain [23,59]. Liver injury has been described also with all triazoles, ranging from mild elevations in transaminases to fatal hepatic failure [60,61,62]. Generally, in most cases of hepatic injury due to triazoles, normalization of the liver enzymes and resolution of the clinical symptoms occurred gradually after the discontinuation of the drug [21,63]. Additionally, triazoles are involved in numerous DDIs because they are substrates and inhibitors of CYP450 isoenzymes [63,64].

#### 2.3.1. Fluconazole

Fluconazole, unlike the other triazoles, is characterized by high water solubility and approximately 60–80% of the drug is eliminated by the kidneys, while hepatic metabolism does not play an important role in the elimination of the drug [29]. The fluconazole dosage regimen for IFIs is guided by the indication, and the daily dose recommended by the manufacturer is up to 400 mg, but in clinical practice it usually ranges from 400 to 800 mg [49,65]. It is well tolerated, even in cases requiring long-term administration of the drug [21]. Nevertheless, up to 10% of patients treated with fluconazole developed asymptomatic liver injury, with those with AIDS or bone marrow transplantation being at greater risk [40,66,67,68,69]. Hepatic injury was typically transient and usually resolved despite drug continuation [21]. Cholestatic and mixed patterns of hepatic injury have been reported, and reinstitution of fluconazole resulted in recurrences in many cases [67,70,71,72]. Furthermore, there are some limited data to suggest that liver injury is dose-related [67,73]. In a large meta-analysis of antifungals tolerability and hepatotoxicity, the risk of liver injury with standard dose of fluconazole not requiring treatment discontinuation was 9.3%, while the risk of drug discontinuation due to elevated liver enzymes was 0.7% [74]. Despite the fact that the risk of acute liver failure due to fluconazole treatment is minimal [74,75], there are some case reports describing deaths attributable to liver dysfunction [66,76,77,78].

Few reports exist regarding the use of fluconazole in patients with pre-existing liver disease. Ruhnke et al. evaluated the PKs of a single 100 mg dose of fluconazole in 9 patients with cirrhosis, classified as group B or group C according to Child-Pugh score, compared with 10 healthy subjects [79]. They found that in cirrhotic patients the terminal elimination constant for fluconazole was lower, and that the total plasma clearance was reduced and the mean residence time increased. The authors assumed that this may be due to kidney dysfunction not reflected in creatinine clearance or the DDIs between fluconazole and diuretics that cirrhotic individuals were receiving. Nevertheless, the authors argued that dosage adjustment of fluconazole in patients with liver impairment is unnecessary, because of the wide range of values they found and the known low toxicity of fluconazole [79]. At the clinical level, Gearhart first described a 50-year old woman with hepatitis who received fluconazole for *Candida* infection and experienced worsening of liver function, which returned to baseline after discontinuation of the drug [80].

A population-based study by Lo Re et al. assessed the risk of acute liver injury with oral azole antifungals in the outpatient setting [81]. Liver aminotransferase levels and development of hepatic dysfunction were examined in 195,334 new initiators of these drugs, for a period of 182 days after the last day’s supply. Fluconazole initiators were 178,879 and, among them, 7073 individuals had pre-existing liver disease. The authors found that the risk of transaminitis (liver aminotransferases > 200 U/L) and severe liver injury [international normalized ratio (INR) ≥ 1.5 and total bilirubin (TB) > 2× ULN] in patients without history of chronic liver disease was lower among users of fluconazole, compared to other azoles. Nevertheless, it should be taken into account that, with the exception of itraconazole, patients administered other azoles were probably of worse health status compared to those administered fluconazole. More interestingly, compared to patients without chronic liver disease who received fluconazole, patients with pre-existing liver disease who were treated with the same drug had higher absolute risk and incidence rate of transaminitis (*p* value interaction < 0.001) and of severe liver injury (*p* value interaction < 0.001) [81]. Whether this observation was due to fluconazole, the natural history of the disease, or both, is unclear [81]. However, no dosage adjustment is provided by the manufacturer for patients with liver impairment, although prescribing information includes a warning that fluconazole should be administered with caution to patients with hepatic dysfunction [65].

#### 2.3.2. Itraconazole

Itraconazole is highly lipophilic, undergoes extensive hepatic metabolism, and is eliminated mostly via feces and urine [29]. It is available as capsule, oral solution, and intravenous formulation [82]. The oral solution has higher bioavailability than capsule formulation, and thus they should not be used interchangeably [83]. The adults recommended by the manufacturer dosage depends on the drug formulation and the indication, usually ranging from 200 mg to 400 mg per day, and doses above 200 mg should be divided [82,83]. However, for the treatment of certain fungal infections, such as blastomycosis and histoplasmosis, doses of 200 mg t.i.d. for 3 days and then 200 mg q.d. or b.i.d. as long-term therapy are recommended, while for coccidioidal meningitis doses up to 800 mg per day can be administrated [84,85,86]. Itraconazole-induced liver injury is not uncommon, and the pattern is typically cholestatic, although hepatocellular injury has been described in cases of acute liver failure [21]. In a large meta-analysis, 31.5% of patients treated with itraconazole developed hepatotoxicity, but a great variability of hepatotoxicity definition was noted in the included studies and many patients may have developed liver injury owing to the underlying IFI itself, limiting the validity of these results [87]. Treatment discontinuation due to itraconazole-induced liver injury was observed in 1.6% of patients [87]. In a more recent meta-analysis, Wang et al. estimated the risk of elevation of liver enzymes not requiring discontinuation of therapy at 17.4% among itraconazole recipients, while the respective risk of treatment discontinuation due to liver injury was 1.5% [74].

The use of itraconazole in patients with liver disease is not well studied. In a PK study, a single 100 mg dose of itraconazole was administered in 12 cirrhotic and 6 healthy individuals [88]. Compared with healthy volunteers, a statistically significant reduction in C_max_ and an increase in the elimination half-time of the drug were observed in patients with cirrhosis. Nevertheless, based on the area under the curve (AUC), cirrhotic and healthy individuals had comparable overall exposure to the drug [88]. In the already mentioned observational study of Lo Re et al., 55 patients with chronic liver disease received itraconazole, and onychomycosis was the most common indication for treatment initiation [81]. Interestingly, none of them developed transaminitis or severe acute liver injury [81]. The fact that, in this study, itraconazole was prescribed mainly for a less severe condition such as onychomycosis and probably in lower doses than those recommended for severe IFIs treatment, may be the reasons for its decreased hepatotoxic potential, compared with what has been observed in other studies which included patients with severe fungal infections and multiple comorbidities. No dose adjustment is available for patients with hepatic impairment, but it is recommended that these patients should be carefully monitored when treated with itraconazole [83]. Apart from the periodic assessment of a patient’s liver enzymes levels while on itraconazole, TDM is generally recommended, in order to assure adequate exposure and to minimize potential toxicities [55,58,82,89,90].

#### 2.3.3. Voriconazole

Voriconazole’s chemical structure is similar to fluconazole, but its spectrum of activity is much broader [48]. It is metabolized by CYP450, mainly CYP2C19, which exhibit significant genetic polymorphism, and it is involved in many DDIs. In addition, recent data suggest that voriconazole metabolism can be inhibited in cases of severe inflammation [91]. It is available as tablet, oral suspension, and intravenous solution [92]. The manufacturer’s recommended dose of intravenous formulation for most IFIs is 6 mg/kg b.i.d. on day 1 as a loading dose, followed by 4 mg/kg b.i.d. as a maintenance dose [92,93]. The oral dose for adult patients is 400 mg b.i.d. on the first day followed by 200 mg b.i.d., while if patient response is inadequate, the maintenance dose may be increased from 200 mg b.i.d. to 300 mg b.i.d. [92,93]. A 50% reduction of both loading and maintenance oral doses is recommended for adult patients with a body weight less than 40 kg [92,93]. The incidence of liver injury in patients treated with voriconazole varies significantly among studies, depending mostly on the characteristics of the study population, while the pattern of liver enzyme abnormality is not uniform [94,95,96,97]. Wang et al. found in their meta-analysis that 19.7% of 881 patients who received voriconazole developed elevation of liver enzymes without the need for treatment discontinuation [74]. A more recent meta-analysis of the utility of voriconazole’s TDM included 11 studies and reported a pooled incidence rate of liver injury among voriconazole recipients at 5.7% [98].

Compared with other triazoles, more data exist regarding the use of voriconazole in patients with underlying hepatic impairment. After a single oral dose of 200 mg of voriconazole in 12 patients with mild to moderate hepatic impairment (Child–Pugh Classes A and B), AUC was 3.2-fold higher than in age and weight matched controls with normal liver function [92]. In an oral multiple-dose PK study, AUC at steady state (AUCτ) was similar in individuals with Child–Pugh Class B cirrhosis given a maintenance dose of 100 mg twice daily and individuals with normal liver function given 200 mg twice daily [99]. Based on the aforementioned data, the medication label of voriconazole recommends that individuals with mild to moderate cirrhosis (Child–Pugh Class A and B) receive the same loading dose as individuals with hepatic function, but half the maintenance dose, while no recommendation is given for individuals with Child–Pugh Class C cirrhosis [92].

In a cohort study of 29 patients with severe liver dysfunction, defined as MELD score > 9, who received at least four doses of voriconazole, a deterioration of hepatic biochemistry was observed in 69% of them [100]. The pattern of the liver injury was mixed; hepatocellular and cholestatic in 45%, 35% and 15% of patients, respectively. None of them developed clinical or laboratory signs of worsening hepatic function. The biochemical parameters returned to baseline levels in all patients after the cessation of voriconazole treatment [100]. Lo Re et al included in their study 97 patients with pre-existing liver disease who received oral voriconazole. Among them, 4 developed ALT or AST > 200 U/L and 2 developed severe liver injury (INR > 1.5 and TB > 2 × ULN), but none of them experienced acute liver failure. Individuals with pre-existing liver disease treated with voriconazole had higher rates of severe liver injury than recipients of voriconazole without underlying hepatic disease [81]. A recent single-center retrospective study compared 6 patients with severe liver cirrhosis (Child–Pugh Class C) who were treated with oral voriconazole based on TDM, with 56 individuals without severe liver cirrhosis who received voriconazole in the recommended dosage for IFIs, also under TDM [101]. The daily maintenance doses of voriconazole of the severe cirrhotic patients were in the range of 50 to 200 mg, with a median daily dose at one-third of the median daily dose of the individuals without severe cirrhosis. The median trough serum concentration of the drug was within recommended levels in both groups of patients. Thus, the authors argued that a dose reduction to about one-third that of the standard maintenance dose is required in patients with Child–Pugh Class C cirrhosis [101].

A multicenter retrospective study aimed to investigate the voriconazole trough concentrations and safety in cirrhotic patients receiving the drug [102]. Seventy-eight patients with Child–Pugh Class B or C cirrhosis who had been treated with voriconazole under TDM were allocated to two groups, according to the dosage regimen they had received. Patients in the first group had received the recommended dosage by the manufacturer or a fixed dose of 200 mg twice daily. Patients in the second group had received a loading dose of 200 mg twice daily on day 1, followed by 100 mg twice daily, or a fixed dose of 100 mg twice daily. The steady-state trough concentration of voriconazole was measured in all patients and its relationship with AEs was analyzed. Voriconazole C_min_ values were significantly different between the two groups, and the proportion of C_min_ higher than the super-therapeutic concentration (defined as 5 mg/L) was 63% in the first group and 28% in the second group of patients. While no statistically significant differences were observed in the incidence of AEs between the two groups, these incidences were considered excessively high (26.5% of patients in the first group and 15.9% of patients in the second group). Interestingly, voriconazole C_min_ between patients with an AE and those without AEs in both groups was similar. However, based on the high C_min_ and incidence of AEs in these patients, both the recommended maintenance dose and halved maintenance dose were considered as inappropriately high [102].

The same authors conducted another study including solely patients with Child–Pugh Class C cirrhosis [103]. Patients were allocated to two groups, according to the dosage schedule of voriconazole’s maintenance dose. The first group included those who received 100 mg of voriconazole twice daily, while the second group included those who received 200 mg of voriconazole once daily. There was no significant difference in voriconazole C_min_ between the two groups. However, the proportion of voriconazole C_min_ higher than the upper limit of therapeutic level (defined again as 5 mg/L) in the first and second groups was 34% and 48%, respectively. The incidence of AEs was 21% in the first group and 27% in the second group, with no statistically significant difference. Further analysis revealed that the increasing C_min_ of voriconazole was associated with increasing incidence of AEs, although no statistical significance was found. It was suggested that in patients with Child–Pugh Class C cirrhosis the halved maintenance dose is probably inappropriate, and that lower dosage should be considered in conjunction with early TDM [103].

Voriconazole TDM is generally recommended because of its highly variable PKs, in order to enhance efficacy, to evaluate therapeutic failure due to possible suboptimal drug exposure, and to avoid associated toxicity due to increased serum drug levels [55,58,104]. It is well established in the literature that an elevated drug’s level in the serum is correlated with increased risk of toxicity [104,105,106]. Thus, voriconazole TDM is of paramount importance in patients with pre-existing liver disease, since the drug is extensively metabolized by the liver and this population is more difficult to tolerate a deterioration of hepatic function due to voriconazole-induced liver injury [101,102,103,107]. Various target trough concentrations associated with efficacy and safety have been reported, and most experts aim for voriconazole trough serum concentration of more than 1–1.5 μg/mL for efficacy but less than 5–6 μg/mL for avoiding toxicity [58,89,98,104].

#### 2.3.4. Posaconazole

Posaconazole’s chemical structure resembles that of itraconazole, but it has a wider antimycotic spectrum [29]. Initially, posaconazole was available only as an oral suspension which displays poor and highly variable absorption [108]. Recently, tablet and intravenous formulations with improved bioavailability were approved [109,110,111]. Posaconazole is metabolized in the liver by UDP-glucuronic-transferase, usually without previous oxidation by CYP450, and is eliminated mainly in the feces and, secondarily, in the urine [112]. Noticeably, posaconazole is a potent inhibitor of CYP3A4, thus clinically relevant DDIs may occur [29]. Regarding IFIs, the adult recommended therapeutic dose for oral suspension is 200 mg q.i.d., while the prophylactic dose is 200 mg t.i.d. [113,114]. In addition, for both tablet and intravenous formulation a loading dose of 300 mg b.i.d. on day 1, followed by a maintenance dose of 300 mg once daily, is recommended as prophylactic as well as therapeutic dosage regimen for several IFIs [113,114]. Liver injury occurs in up to 25% of patients receiving posaconazole regardless of the formulation, but this may be multifactorial and not only attributable to the drug [81,115,116,117,118]. The dominant pattern of hepatic injury varies among studies, partly depending on the studied population [21,115,116,117]. In addition, hepatic failure due to posaconazole treatment is generally uncommon [81,110,111,115,116,117].

Regarding the use of posaconazole in individuals with pre-existing hepatic impairment, Moton et al. conducted a PK study to evaluate the need of posaconazole dose adjustment in this population [119]. In their single-center study, the researchers aimed to compare the PKs of a single dose 400 mg of posaconazole oral suspension in 19 patients with varying degrees of hepatic dysfunction with 18 matched healthy individuals who received the same regimen. No clear trend was observed of an increase or decrease in posaconazole exposure linked with increasing degrees of hepatic dysfunction. The detected differences of PKs between healthy individuals and those with hepatic dysfunction were not clinically significant, and the authors suggested that posaconazole dosage adjustment may not be required in individuals with hepatic impairment [119]. A case-report also described a patient with Child–Pugh Class B cirrhosis suffering from maxillary mucormycosis who, after surgical debridement and initial treatment with AmB followed by itraconazole, was successfully treated with oral posaconazole suspension 400 mg twice daily for nine months without hepatic decompensation [120]. In addition, Lo Re et al included in their observational study 9 patients with chronic liver disease who received posaconazole, and only one of them developed severe acute liver injury (INR > 1.5 and TB > 2× ULN) [81].

In a recent single-center retrospective cohort study, Tverdek et al. assessed the real-life safety and effectiveness of primary antifungal prophylaxis with new tablet and intravenous posaconazole formulations in high-risk patients with leukemia and/or hematopoietic stem cell transplantation (HSCT) [116]. A total of 343 patients were included, 62% of whom received 300 mg of posaconazole twice daily on day 1, while 99% received the maintenance dose of 300 mg per day. Among them, 316 patients had baseline liver assessment, including 144 patients with baseline elevations of ALT, ALP, or/and TB, of which 23 had grade 3 or 4 liver injury [121]. Concerning the 121 patients with baseline liver injury but no grade 3 or 4 abnormalities, 34 (28%) of them developed grade 3 or 4 liver injury. Liver abnormalities were developed in nearly 20% of all patients, primarily manifested as hyperbilirubinemia. These abnormalities were more frequent in individuals with pre-existing liver injury, but this may not be solely due to DILI, as the underlying disease and concomitant drugs may also have contributed [116].

Noticeably, in patients with new-onset hepatotoxicity due to voriconazole administration for IFIs, sequential use of posaconazole seems to be safe and effective, with favorable outcomes and improvement of liver biochemistry in most of the cases [122,123,124]. Independently of the acute or chronic nature of pre-existing liver injury, no dosage adjustments are recommended for individuals with hepatic impairment treated with posaconazole [113]. In addition, while many guidelines recommend TDM in patients receiving posaconazole oral suspension for IFI prophylaxis or treatment to confirm adequate absorption and ensure efficacy [58,89], PK/PD analyses conducted with oral posaconazole suspension do not support a relationship between plasma concentrations and toxicity [125,126]. On the contrary, Tverdek et al identified a potential association between elevated serum posaconazole levels and hepatotoxicity in patients treated with the new tablet and intravenous formulations of the drug, but further evaluation is needed [116].

#### 2.3.5. Isavuconazole

Isavuconazole is the newest member of triazoles antifungals. In both oral and intravenous formulations, it is administered as a water-soluble prodrug, isavuconazonium sulfate [127]. After intravenous administration, the prodrug is rapidly hydrolyzed to isavuconazole by plasma esterases, while oral formulation of isavuconazonium sulfate sustains chemical hydrolysis in the gastrointestinal lumen [112]. Metabolism of isavuconazole takes place in the liver by CYP450 isoenzymes, with subsequent glucuronidation by uridine diphosphate-glucuronosyl transferase (UGT) [127]. Isavuconazole is generally well tolerated and safe, and has fewer DDIs compared with voriconazole and posaconazole, but clinical experience is still limited [60,61]. It is approved by the FDA and the European Medicine Agency (EMA) for the treatment of adult patients with invasive aspergillosis or invasive mucormycosis, with a loading dose of 200 mg t.i.d. for the first two days, followed by a maintenance dose of 200 mg q.d., via oral or intravenous administration [127,128]. Elevations in liver enzymes have been reported in clinical trials but they are generally reversible and rarely only require treatment discontinuation [129,130,131]. However, cases of severe liver injury have occurred during treatment with this antifungal agent [127,129]. In a phase 3 comparative study evaluating isavuconazole versus voriconazole for the treatment of invasive aspergillosis, there were significantly higher liver disorders in the voriconazole arm (*p* value = 0.016), but the protocol of the study did not allow TDM [131]. Since voriconazole displays highly variable non-linear pharmacokinetics in adults and, thus, TDM is recommended, these results should be interpreted with caution, and further research is needed.

An initial single-dose PK study aimed to assess the effect of mild to moderate hepatic impairment due to alcoholic cirrhosis on the disposition of isavuconazole [132]. Clearance values of isavuconazole were significantly decreased and half-life values were significantly increased in cirrhotic patients compared with healthy individuals, leading the authors to recommend a 50% decrease in the maintenance dose of the drug for patients with mild or moderate liver disease [132]. However, a subsequent population PK analysis used data from the aforementioned study and from another study and reported different results [133]. The PK and safety results showed that dose adjustment appears to be unnecessary for patients with Child–Pugh Class A or Class B cirrhosis treated with isavuconazole, since there was a less than twofold increase in trough concentrations for those compared with healthy subjects, while the AEs profile was similar between cirrhotic and healthy individuals [133].

Notwithstanding, both these PK studies did not take PD into consideration, which may affect the dose of isavuconazole against different fungi in this population of patients. In a recently published PK/PD study, Zheng et al. examined the efficacy of various isavuconazole dosing regimens for healthy individuals and patients with renal and hepatic impairment, namely Child-Pugh Class A or B cirrhosis, against *Aspergillus* spp. and other fungi [134]. The Monte Carlo simulation was used in each scenario to calculate target attainment and cumulative fractions of response probabilities. The clinically recommended dose of 200 mg isavuconazole per day was effective for all individuals against *A. fumigatus*, *A. flavus*, *A. nidulans*, *A. terreus*, and *A. versicolor*. [134].

In the manufacturer’s labeling, the standard dose of isavuconazole is recommended for patients with mild or moderate liver dysfunction, while the drug has not been studied in patients with Child–Pugh Class C hepatic impairment, and should be used in these individuals only when the benefits outweigh the risks [127]. Although TDM of isavuconazole may be considered in selected patients, such as those with severe hepatic impairment, routine TDM for isavuconazole is not recommended [135].

### 2.4. Echinocandins

Echinocandins inhibit the synthesis of 1,3-β-d-glucan, a fungal cell wall component, resulting in instability of the cell wall, cell lysis, and death [136]. The fact that this class of antifungals agents targets the fungal cell wall and not the cell membrane explains the absence of cross-reactivity with mammalian cells and the excellent tolerability of this class of compounds in humans [48]. They are fungicidal to *Candida*, including several non-albicans strains, and fungistatic to *Aspergilli,* thus they are considered the first-line treatment for *Candida* spp. infections [29,49]. At present, the available agents of this class include caspofungin, micafungin, and anidulafungin [23]. Common AEs related with echinocandins treatment include phlebitis, nausea, diarrhea, headache and pruritus, but also other drug reactions such as leukopenia, anemia, hypokalemia, and liver injury have been reported [29]. Noticeably, the echinocandins have less than half the likelihood of discontinuation of therapy due to AEs, compared with triazoles [137].

#### 2.4.1. Caspofungin

Caspofungin bounds to plasma proteins at 95%; it is transformed in the liver but only minimally undergoes degradation by CYP450 isoenzymes, and the metabolites are eliminated via urine [138,139]. The recommended dosage for adults is 70 mg as a single loading dose on day 1, followed by a maintenance dose of 50 mg once daily [140,141]. The EMA recommends an increase of maintenance dose to 70 mg daily when patient’s body weight exceeds 80 kg [140]. Generally, hepatic abnormalities related to caspofungin treatment are uncommon and severe hepatic AEs are rare [21]. In most studies, elevated hepatic enzymes were observed in up to 9% of patients, and they were often clinically irrelevant [24].

Regarding patients with pre-existing liver disease treated with caspofungin, Mistry et al. conducted single- and multiple-dose open-label studies to assess dosage and safety of caspofungin in hepatic impairment [142]. Patients with Child–Pugh score 5–6 or 7–9 hepatic impairment were matched with healthy individuals. Patients with Child–Pugh score 5–6 hepatic impairment had a mild elevation in caspofungin serum concentration, which was considered as clinically irrelevant. Patients with Child–Pugh score 7–9 hepatic impairment needed a reduced maintenance dose of caspofungin in order to achieve drug concentrations similar with the healthy individuals in the control group [142]. Based mainly on these data, a reduction of caspofungin maintenance dose from 50 mg to 35 mg per day is recommended for patients with Child–Pugh Class 7–9 hepatic impairment, while no recommendation is given for patients with Child–Pugh score 10–15 hepatic impairment [141].

However, Spriet et al. initially described a patient with Child-Pugh Score 9 cirrhosis diagnosed with acute myeloid leukemia, who was treated for a severe IFI with a full dose of caspofungin 70 mg per day, since his body weight was over 80 kg [143]. The PK data of this case-report indicated that if the reduced dose of caspofungin had been used, it would probably have resulted in a low caspofungin systemic exposure and a possible therapeutic failure [143]. A subsequent population PK analysis concluded that a reduction of caspofungin maintenance dose in non-cirrhotic intensive-care unit (ICU) patients, who are misclassified due to hypoalbuminemia as with Child–Pugh Class B hepatic impairment, is not recommended, because it may result in significantly lower drug exposure and possible therapeutic failure [144]. On the contrary, authors suggested that, depending on pathogens MIC, a caspofungin maintenance dose of 70–100 mg daily may be reasonable in many cases [144].

Furthermore, data from the aforementioned population PK analysis in non-cirrhotic ICU patients were used in another PK study of a single-dose of 70 mg of caspofungin in patients with decompensated Child–Pugh Class B or C cirrhosis to evaluate the impact of cirrhosis and hepatic impairment severity on the PK of the drug [145]. Remarkably, their data showed that cirrhosis had a limited impact on clearance of caspofungin. Also, it was the first study providing PK data of caspofungin for patients with Child–Pugh Class C cirrhosis and compared with patients with Child–Pugh Class B cirrhosis, no further decrease of caspofungin clearance was observed in the former group of individuals. Thus, the researchers concluded that reducing the dose of caspofungin in patients with Child–Pugh Class B or C cirrhosis leads to a decrease in exposure and this may result in a suboptimal clinical outcome [145]. In another recent PK study for general patients, ICU patients, and patients with hepatic impairment receiving caspofungin, a whole-body physiology-based PK model was developed and was combined with Monte Carlo stimulation to optimize dosage regimen of the drug in patients with different characteristics [146]. The results of this study indicated that the caspofungin maintenance dose should not be reduced to 35 mg per day for ICU patients classified as Child–Pugh Class B when this classification is driven by hypoalbuminemia, as lower drug exposure occurs. On the contrary, authors argued that, in any other case, a reduction of caspofungin maintenance dose to 35 mg per day for patients with moderate hepatic impairment classified as Child–Pugh Class B, may be reasonable [146].

#### 2.4.2. Micafungin

Micafungin is highly bound to proteins, it is metabolized in the liver by enzymes unrelated to CYP450, and the metabolites are excreted primarily via feces [147]. The recommended dosage for patients weighing greater than 40 kg is 100 once daily for the treatment of invasive candidiasis, and 150 mg once daily for the treatment of *Candida* esophagitis [148,149]. It is a well-tolerated antifungal agent with few AEs requiring cessation of the drug [21]. Mild elevations of hepatic enzymes may occur, but clinically overt liver toxicity is rare [23,150]. Nevertheless, rat models demonstrated an association between micafungin and foci of altered hepatocytes and hepatocellular tumors when this was given for more than 3 months, but this finding has not been replicated in humans [23,29].

Micafungin has a low hepatic extraction ratio with high protein binding in plasma, and while its total plasma concentration may decrease in some clinical cases, the unbound fraction of the drug is likely to remain stable [151,152]. A phase I parallel group open-label PK study of a single-dose of micafungin included 8 patients with Child–Pugh Score 7–9 hepatic dysfunction and did not find significant difference in unbound plasma concentration of the drug compared with healthy controls, while a lower AUC was found in the patients with hepatic impairment [153]. The latter was attributed to the differences in body weight among patients, and no dose adjustment was recommended [153]. In an another open-label single-dose PK study, 8 patients with Child–Pugh score 10-12 hepatic impairment and 8 healthy individuals received 100 mg of micafungin [154]. Compared with healthy subjects, patients with hepatic dysfunction had lower C_max_ and AUC values, but the magnitude of differences was considered as clinically meaningless and no dose reduction was recommended in patients with severe hepatic impairment [154]. In addition, Luque et al. conducted a prospective observational study to assess the possibility of DILI due to micafungin use in daily practice including 12 patients, 8 of whom had elevated liver enzymes at the beginning of the treatment [155]. The daily dose of micafungin was 100 mg for 10 patients and 150 mg for the remaining two. There was no correlation between the degree of the pre-existing liver injury and micafungin levels. In steady state, C_max_ and C_min_ were similar in subjects with and without initial liver abnormalities. Hepatic enzymes levels remained stable or even improved in all but one patient. These results further support the safety of micafungin in patients with pre-existing liver injury and IFIs [155]. Based on most of the aforementioned studies, the summary of manufacturers’ product characteristics approved by the FDA recommends that no dosage adjustment is required in patients with hepatic impairment [149]. Contrarily, EMA recommends avoidance of micafungin use in patients with severe hepatic impairment, while it has issued a black-box warning for hepatotoxicity and potential for liver tumors [148].

#### 2.4.3. Anidulafungin

Anidulafungin has a very high protein binding of 99%; it is degraded non-hepatically in the blood, and the metabolites are eliminated via feces [156]. The recommended adult dosage for invasive candidiasis is a single loading dose of 200 mg on day 1, followed by a maintenance dose of 100 mg once daily [157,158]. Anidulafungin AEs, including DILI, are generally infrequent [159,160]. With regard to patients with pre-existing hepatic disease treated with this antifungal agent, Dowel et al. conducted a phase I, open-label, single-dose study including 20 patients with varying degrees of hepatic impairment and 7 healthy controls [161]. No statistically significant differences in PK parameters were observed between healthy controls and patients with mild or moderate hepatic impairment. However, compared with healthy controls, subjects with severe hepatic impairment (Child–Pugh Class C) showed statistically significant decreases in C_max_ and AUC values, most likely secondary to ascites and edema, but anidulafungin exposure remained significantly above MIC_90_ of many common fungal pathogens. Additionally, the values of all PK parameters still remained within the range that had been previously reported in healthy subjects. No evidence of dose-depended toxicity or serious AEs was observed. Thus, the authors suggested that anidulafungin can be safely used in patients with hepatic dysfunction without dosage adjustment [161].

In a retrospective cohort study, Verma et al. assessed the safety and efficacy of anidulafungin in the treatment of IFIs in patients with hepatic impairment or multiorgan failure [162]. Fifty patients were included, among them 30 with a calculated baseline MELD score, of whom 13 had a score ≥ 30. A dose of 200 mg was given to all patients on day 1, followed by 100 mg per day onwards. Before initiation of treatment with anidulafungin, at least one abnormal liver function test (LFT) was observed in 49 of 50 patients (98%). During treatment, LFTs worsened in many patients, but fewer patients had elevated LFTs at the completion of treatment than at the beginning. A favorable outcome was seen in more than 75% of patients. The latter further supports indications that anidulafungin is efficacious and safe in patients with decompensated hepatic disease and, in agreement with package insert recommendations, no dosage reduction is needed in patients with any degree of hepatic impairment [157,162].

## 3. Clinical Implications and Future Directions

Patients treated with antifungal agents for IFIs may have underlying hepatic impairment of varying degrees and origin. Clinicians should be aware of that, since it further complicates management with regard to efficacy and safety of the antifungal therapy. Firstly, metabolism and elimination of many antifungals are significantly altered by hepatic dysfunction, while DDIs are somewhat unpredictable compared to individuals with intact liver function. Moreover, it may be difficult to attribute further deterioration of liver biochemistry or function only to antifungals in patients with severe comorbidities and concomitant administration of other hepatotoxic drugs. In addition, precise estimates of hepatic function are currently unavailable. The Child–Pugh system, on which most dosage modifications in hepatic impairment are based, was initially developed to assess the prognosis of chronic liver disease and not the degree of hepatic dysfunction [20]. For all the above reasons, the optimal use of antifungals in patients with pre-existing liver disease with IFIs is still unfolding. Data discussed in the present review give rise to useful clinical suggestions for the optimization of treatment. Table 2 summarizes the dosage adjustments of antifungal agents that are approved and recommended by FDA and/or EMA for patients with hepatic impairment treated for IFIs, and also presents the recommendations included in many guidelines regarding TDM of certain antifungal drugs for optimizing efficacy and safety.

With regard to AmB, to date few data exist on the necessity for dosage adjustment of any AmB formulations in patients with hepatic impairment. However, the lipid formulations of the drug seem to have a higher potential for hepatotoxicity compared to AmBD. In addition, AmB formulations combined with flucytosine for the treatment of certain fungal infections may lead to increased flucytosine serum levels due to kidney injury and accumulation of the renally eliminated drug. Flucytosine TDM is of clinical importance generally, in order to assure efficacy and to prevent AEs, including hepatotoxicity.

Fluconazole dosage modification for hepatic impairment per se is not required. Nevertheless, it should be used cautiously in this subset of patients due to the increased risk of further deterioration of hepatic enzymes levels and/or hepatic function compared to subjects with normal liver function. For itraconazole there are no dosage adjustment recommendations available for patients with hepatic dysfunction, however its use is discouraged in this subset of patients unless benefit exceeds risk. In the latter case, close monitoring, including TDM, is recommended, but further work is necessary for establishing clear drug target levels.

Use of voriconazole has also an increased risk for severe live injury in patients with chronic liver disease. While reduction of voriconazole’s maintenance dose by 50% is recommended in patients with Child–Pugh Class A or B cirrhosis, data for patients with more severe hepatic impairment were lacking until recently. New evidence suggests that dose should be lowered more than 50% in patients with Child–Pugh Class C hepatic dysfunction, and always under TDM for safety and efficacy enhancement [101,102,103,163]. However, optimal dosage in this setting has not formally been defined and this is a noteworthy area of active research. Likewise, posaconazole and isavuconazole have not been studied sufficiently in patients with severe hepatic impairment and more research on that topic is of paramount importance. Furthermore, only recently a possible relationship between increased posaconazole serum levels and liver toxicity was identified in patients receiving the new intravenous and tablet drug formulations, thus more PK studies are needed, especially in patients with underlying liver disease [116]. Regarding isavuconazole, generally it demonstrates a favorable safety profile in relation to DDIs and hepatotoxicity. Nevertheless, compared with other triazoles, published clinical experience and post-marketing data, including its use in special patient populations, are still limited.

Compared with triazoles, echinocandin use in patients with underlying hepatic impairment is considered relatively safe. A reduction to caspofungin maintenance dose is recommended for patients classified with Child–Pugh Score 7–9 hepatic dysfunction, yet this has been challenged recently and clinicians should be aware of that, since it may result in suboptimal exposure in critically ill patients [144,145,146]. With regard to micafungin, no dosage modification is recommended in mild and moderate hepatic insufficiency, but additional research seems necessary for patients with severe hepatic impairment. Among this class of antifungal agents, anidulafungin may have an advantage for use in cirrhotic patients due to its non-hepatic metabolism, more predictable PK, and favorable tolerability. However, this remains to be further evaluated with future comparative studies in this subset of patients.

## 4. Conclusions

Treatment of IFIs in patients with pre-existing liver disease poses a significant challenge for clinicians. These patients are often more vulnerable to the hepatotoxic potential of many antifungal agents, while possible alterations of the PKs of these drugs may trigger adverse effects not localized only to the liver. Current evidence from PK studies and safety data from the existing clinical trials and post-marketing studies can help physicians optimize IFIs treatment in this special group of patients. However, most of the existing evidence is limited to subjects with mild to moderate hepatic disease, and clear recommendations for dosage adjustments in cases of severe hepatic impairment are not yet available for the majority of antifungal agents. This raises the need for more PK and clinical studies in this subset of patients. Furthermore, additional attention should be paid to future pharmacovigilance monitoring of antifungal agent use in patients with liver disease of any degree. In any case, close clinical and laboratory monitoring, including TDM for specific antifungal drugs, is essential in the majority of these patients in order to prevent or promptly recognize further deterioration of the hepatic function, thus avoiding unfavorable outcomes.

## Figures and Tables

**Table 1:** Clinical chemistry criteria for DILI.

Anyone of the Following *:
ALT elevation ≥ 5 × ULN ^¶^
ALP elevation ≥ 2 × ULN ^¶^, especially with accompanying elevations in concentrations of 5′-NT or GGT
ALT elevation ≥ 3 × ULN ^¶^ and simultaneous TB elevation ≥ 2 × ULN ^¶^

DILI: drug-induced liver injury; ALT: alanine transaminase; ULN: upper limit of normal; AST: aspartate transaminase; ALP: alkaline phosphatase; 5′-NT: 5′-nucleotidase; GGT: γ-glutamyl transpeptidase; TB: total bilirubin. * After other causes have been ruled-out [15]. ^¶^ In cases of pre-existing abnormal biochemistry before the administration of the implicated drug, ULN is replaced by the mean baseline values obtained prior to drug exposure [15].

**Table 2:** Antifungal agent dosage adjustment for patients with hepatic impairment.

Antifungal Agent	Severity of Hepatic Impairment by Child–Pugh Score		
	Score 5–6 (Class A)	Score 7–9 (Class B)	Score 10–15 (Class C)
AmB preparations	No recommendations available		
Flucytosine	No recommendations available, use with caution, TDM recommended Authors’ comment: extra caution when combined with AmB preparations		
Fluconazole	No recommendations available, use with caution		
Itraconazole	No recommendations available, strongly discouraged unless benefit exceeds risk, use with caution and under close monitoring, TDM is recommended		
Voriconazole	50% reduction of maintenance dosage and TDM are recommended		No recommendations available, use only if benefit outweighs risk, close monitoring and TDM are recommended Authors’ comment: reduction of maintenance dosage to about one-third may be considered
Posaconazole	No dosage adjustment is recommended, TDM when oral suspension is used Authors’ comment: TDM may also be considered when tablet or intravenous drug formulation is used		
Isavuconazole	No dosage adjustment is recommended		No recommendations available, use only if benefit outweighs risk
Caspofungin	No dosage adjustment is recommended	Reduced maintenance dose from 50 mg to 35 mg daily Authors’ comment: in critically ill patients, reduced dosage may lead to decreased drug exposure	No recommendations available
Micafungin	No dosage adjustment is recommended		US FDA recommends no dosage adjustment, EMA recommends avoidance of its use
Anidulafungin	No dosage adjustment is recommended		

AmB: amphotericin B; TDM: therapeutic drug monitoring; US FDA: United States Food and Drug Administration; EMA: European Medicines Agency.
